# Genetic Characterization of Five Hatchery Populations of the Pacific Abalone (*Haliotis discus hannai*) Using Microsatellite Markers

**DOI:** 10.3390/ijms12084836

**Published:** 2011-07-29

**Authors:** Hye Suck An, Jang Wook Lee, Hyun Chul Kim, Jeong-In Myeong

**Affiliations:** Genetics and Breeding Research Center, National Fisheries Research and Development Institute, Gyeongsangnamdo 656-842, Korea; E-Mails: lee9952@nfrdi.go.kr (J.W.L.); kimhc@nfrdi.go.kr (H.C.K.); cosmo@nfrdi.go.kr (J.-I.M.)

**Keywords:** Pacific abalone, *Haliotis discus hannai*, genetic structure, hatchery populations, microsatellite loci

## Abstract

The Pacific abalone, *Haliotis discus hannai*, is a popular food in Eastern Asia. Aquacultural production of this species has increased because of recent resource declines, the growing consumption, and ongoing government-operated stock release programs. Therefore, the genetic characterization of hatchery populations is necessary to maintain the genetic diversity of this species and to develop more effective aquaculture practices. We analyzed the genetic structures of five cultured populations in Korea using six microsatellite markers. The number of alleles per locus ranged from 15 to 64, with an average of 23.5. The mean observed and expected heterozygosities were 0.797 and 0.904, respectively. The inbreeding coefficient *F*_IS_ ranged from 0.054 to 0.184 (mean *F*_IS_ = 0.121 ± 0.056). The genetic differentiation across all populations was low but significant (overall *F*_ST_ = 0.009, *P* < 0.01). Pairwise multilocus *F*_ST_ tests, estimates of genetic distance, and phylogenetic and principal component analyses did not show a consistent relationship between geographic and genetic distances. These results could reflect extensive aquaculture, the exchange of breeds and eggs between hatcheries and/or genetic drift due to intensive breeding practices. Thus, for optimal resource management, the genetic variation of hatchery stocks should be monitored and inbreeding controlled within the abalone stocks that are being released every year. This genetic information will be useful for the management of both *H. discus hannai* fisheries and the aquaculture industry.

## 1. Introduction

The abalone is a marine gastropod of the genus *Haliotis*. Nearly 100 Haliotis species inhabit both tropical and temperate waters worldwide [1]. Among the more than 30 species of commercially available abalone, the Pacific abalone (*Haliotis discus hannai*), which is distributed in the coastal areas of Eastern Asia, including Korea, Japan, and China, is one of the most popular species [2]. In response to a decrease in natural resources in the face of increasing demand, the aquacultural production of abalones has increased [3]. Culturing of *H. discus hannai* on a mass scale in Korea began in the 1980s, and has rapidly developed in the past decade. The majority of the annual yield now comes from aquaculture. The annual production of cultured *H. discus hannai* reached approximately 7500 tons in 2009 [4]. In addition to increased abalone farming, the Korean government has sponsored artificial abalone seed release for coastal abalone resource enhancement since 1997. More than five million Pacific abalone seeds reared in hatcheries were released into the Korean coastal sea areas in 2007 [5].

Natural populations of *H. discus hannai* have been overexploited to the extent that wild Pacific abalone is difficult to obtain in Korea. Pacific abalone seed is usually produced in hatcheries using reared adults as broodstock. Hence, hatchery production of abalone raises concerns regarding the maintenance of genetic diversity among cultured stocks, especially because their seedlings are released into natural habitats and thus could potentially alter the genetic structure of natural populations [6]. Despite a long history of aquaculture in Korea, the genetic diversity of hatchery stocks remains unknown. Therefore, an investigation of genetic variation in cultured abalone stocks is urgently needed for successful hatchery management, the production of high-quality abalone and to avoid reductions to the genetic variation present in aquaculture stocks.

The monitoring of genetic variation among marine resources, especially in species for which artificial stocks produced by aquaculture are used for the restoration of natural resources, is essential to ensure that stock enhancement programs successfully preserve genetic diversity. This monitoring necessitates the development of genetic markers that can be used to assess genetic variation among populations and to prevent the release of mixed hatchery seeds, which cannot be detected by eye.

Among available genetic markers, microsatellites are recognized as an essential tool in population studies because of their useful properties, such as high levels of polymorphism, codominant inheritance, and good reproducibility [7,8]. Over the past decade, microsatellites have produced promising results in studies of genetic variation in many marine species [9–13].

Until recently, microsatellite markers have been developed in the Pacific abalone [14–18], and genetic variability of hatchery stocks in Pacific abalone has been analyzed [6,17–20]. Microsatellite markers could sensitively detect the reductions of genetic variability on allelic diversity and mean heterozygosity. Highly significant *F*_ST_ values were observed between hatchery stocks in Japan and China [6,19,20]. There are only two examinations for genetic characterization of new microsatellite markers from Pacific abalone in Korea and they reported microsatellite markers could detect significant differentiation between one wild population and one hatchery population [17,18].

In the present study, we used six microsatellite markers to analyze the genetic diversity and relationships within and between cultured populations of *H. discus hannai* from different regions in Korea. This study will provide useful data for the effective monitoring and management of abalone populations as well as for the implementation of a stock enhancement program.

## 2. Materials and Methods

### 2.1. Sample Collection and DNA Extraction

**Figure 1.**
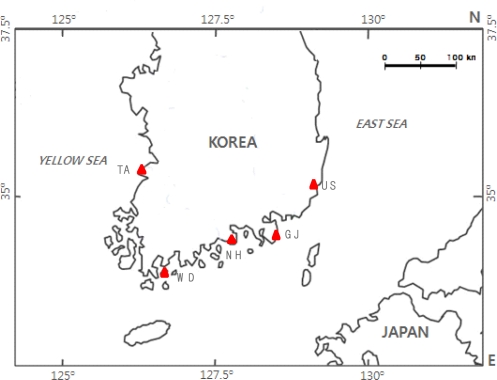
Sampling sites and abbreviated names of five hatchery-reared Pacific abalone (*H. discus hannai*) samples (▴) in Korea. The abbreviations are as follow: US (Ulsan), GJ (Geoje), NH (Namhae), WD (Wando) and TA (Taean).

For the analysis, 223 Pacific abalones (*H. discus hannai*) were collected from five coastal locations in Korea in 2003 and 2004 (Figure 1). To cover the main coastal areas in Korea, five hatchery populations were sampled from three different areas (Table 1). The samples were obtained from hatchery-reared populations used as broodstock for artificial reproduction in Ulsan, in the eastern coastal areas (US; 51 individuals); Namhae (NH; 49 individuals) and Wando (WD; 41 individuals) in the southern coastal areas; and Taean (TA; 39 individuals) in the western coastal areas. The samples for one hatchery-reared population were acquired from a population of hatchery-reared offspring in Geoje (GJ; 43 individuals) in the southern coastal areas. The WD hatchery managed a large broodstock composed of wild-caught and hatchery-produced adult Pacific abalones, the GJ hatchery kept no broodstock but has used hatchery-produced adults as parents for reproduction, and the others had small broodstocks composed of hatchery-produced adults. Although the original ancestors of the hatchery abalone were collected locally and the hatchery populations had been reared continuously, no detailed records of their founding and maintenance were available.

Mantle musculature clips were preserved in 99.9% ethanol before being transported to the lab. Total DNA was extracted using the automated DNA extraction system MagExtractor MFX–2100 (Toyobo) with a MagExtractor-Genomic DNA Purification Kit (Toyobo, Osaka, Japan). Extracted genomic DNA was stored at −20 °C prior to polymerase chain reaction (PCR) analysis.

### 2.2. Microsatellite Genotyping

In total, 223 Korean Pacific abalones from the five populations were genotyped using six Pacific abalone microsatellite loci. Hdh1321, Hdh513, Hdh512 and Hdh145 developed for *Haliotis discus hannai* [15] and Hdd114B and Hdd229 developed for *Haliotis discus discus* [14] were used to amplify alleles by PCR. The 5’-end of the forward primer of each set of primers was labeled with fluorescent dye (6-FAM, HEX, or NED; Applied Biosystems, Foster City, CA, USA). PCR amplification of the six microsatellite loci was performed in 10-μL volumes containing 0.25 U Taq DNA polymerase, 10× ExTaq buffer, 2 mM dNTP mixture (Takara, Shiga, Japan), 2 μM of each primer set and approximately 10 to 50 ng template DNA using a PTC-0220 DNA Engine Dyad Peltier thermal cycler (MJ Research, Inc., Waltham, MA, USA). PCR conditions included an initial denaturation at 95 °C for 11 min, followed by 35 cycles of denaturation at 94 °C for 1 min, annealing for 1 min at each primer temperature listed in Table 1, and extension at 72 °C for 1 min, with a final extension at 72 °C for 5 min.

For genotyping, 1 μL of PCR product was added to 9 μL of a reaction containing formamide (Hi-Di Formamide, Applied Biosystems, Warrington, UK) and the GeneScan 400HD [ROX] size standard (ABI PRISM, Applied Biosystems, CA, USA), denatured at 95 °C for 2 min, and immediately chilled on ice. Fragment analysis of the reaction products was performed using an ABI 3130 Genetic Analyzer (Applied Biosystems) and GeneMapper software (ver. 4.0; Applied Biosystems). To improve accuracy when determining allele sizes, a control DNA sample was included in each set of samples for each run.

### 2.3. Data Analysis

Statistical genetic analyses were conducted for five populations of *H. discus hannai*. The data were tested for PCR errors due to null alleles, stuttering, and allele dropout using MICRO-CHECKER ver. 2.2.3 (1000 randomizations) [21]. Scoring and human error were estimated by duplicate analyses. The data were also tested whether one or more microsatellites were under selection using LOSITAN software [22,23], which is a selection detection workbench based on a well evaluated *F*_ST_-outlier detection method. Two genetic diversity parameters, the number of alleles per locus (*N*_A_) and the number of unique alleles (U), were determined for each local sample at each locus, using the program Genepop ver. 4.0 (http://kimura.univ-montp2.fr/~rousset/Genepop.htm). Allelic richness (*A*_R_) was corrected for the smallest sample size (*n* = 39), using the rarefaction method of FSTAT ver. 2.9.3.2 [24]. Using FSTAT, allelic richness can be directly compared among populations, regardless of sample size [25]. For the analysis of molecular variance (AMOVA) [26], components of variance within and between populations based on the infinite allele model (IAM) were estimated using the program Arlequin ver. 3.0 [27]. The significance of AMOVA components was tested using 1,000 permutations. To estimate genetic heterozygosity among the entire set of pairwise population samples, unbiased expected (*H_e_*) and observed (*H_o_*) heterozygosity values [28] were calculated. Overall inbreeding coefficients (*F*_IS_) [29] for each population and locus were also estimated; *F*_IS_ can measure deviations from the Hardy–Weinberg equilibrium (HWE) within a population. Deviations from the HWE were tested using probability tests or exact tests by the Markov-chain procedure of Arlequin.

The extent of population subdivision was examined by calculating global multilocus *F*_ST_ values [29] and *R*_ST_ values (1000 permutations) [30]. The index of pairwise *F*_ST_ based on an infinite alleles model (IAM) was estimated and *R*_ST_ values based on a stepwise mutation model (SMM) were calculated using Arlequin. The index *R*_ST_ incorporates the correlation of the weighted mean allele size, expressed as the number of tandem repeats. Significance levels were adjusted for multiple tests using the sequential Bonferroni correction technique [31].

The genetic distance between populations was estimated on the basis of the chord distance, *D*_CE_ [32]. *D*_CE_ is one of the most efficient distance measures in obtaining correct tree topology from allele frequency data [33]. To examine genetic relationships between populations, a phylogenetic tree was constructed on the basis of pairwise genetic distances for all samples using the UPGMA method of the program POPULATIONS ver. 1.2.30 (http://bioinformatics.org/~tryphon/populations/). Bootstrap values were calculated using 1,000 replicates. The UPGMA tree was visualized using the Tree Explorer program (http://evolgen.biol.metro-u.ac.jp.TE/).

Relationships between geographical populations were assessed using principal component analysis (PCA) of GenAlEx 6.3 (http://www.anu.edu.au/BoZo/GenAlEx/), which was based on the covariance matrix of gene frequencies.

Because hatchery populations are often subjected to founder effects and bottlenecks that result in lower genetic diversity, Bottleneck software ver. 1.2.02 [34] was employed to test the bottleneck hypothesis under a two-phased model of mutation (TPM). This method can be used for testing the departure from mutation-drift equilibrium based on heterozygosity excess or deficiency.

## 3. Results

### 3.1. Genetic Variability

Micro-Checker analysis did not detect any allele scoring errors caused by stuttering or large allele dropout, but it indicated that five of six loci could carry null alleles (Hdd114B was not affected; *P* < 0.05). However, all six loci were used in this study because no null alleles affected all populations.

Lositan analysis did not detect any outlier loci that had excessively high or low *F*_ST_ compared to neutral expectations, indicating all microsatellites used were not candidates for being subject to selection.

Estimates of genetic variability for the five Pacific abalone populations are summarized in Table 2. These results suggest that all of the microsatellite loci were polymorphic, with some level of polymorphism, including large differences in the number of alleles, in all of the studied populations. N_A_ and A_R_ ranged from 20.17 in the TA population to 26.67 in NH and from 20.17 in TA to 24.78 in NH, respectively, with the degree of variability differing among the six loci. The mean expected and observed heterozygosities per locus ranged from 0.885 in WD to 0.919 in NH and from 0.752 in NH to 0.838 in WD, respectively. However, despite these differences in genetic diversity, no significant decrease in genetic variability was found among the population samples (Kruskal-Wallis test, *P* > 0.05). In total, 56 alleles were found to be unique to a single population. The greatest number of unique alleles was detected in NH (20), while TA had only seven unique alleles. The inbreeding coefficient *F*_IS_ of the hatchery populations ranged from 0.054 to 0.184 (mean *F*_IS_ = 0.121 ± 0.056).

The observed genotype frequencies were tested for agreement with HWE (Table 2). Among the 30 population-locus cases, in 18 cases the observed genotype distribution was generally in accordance with Hardy-Weinberg proportions; however, 12 (40%) departed significantly from HWE, even after adjustment of *P* values using the sequential Bonferroni method (*P* < 0.01), and these disequilibriums (DHWE) were detected at all loci except Hdh114B. Ten DHWE cases resulted from a deficiency of heterozygotes and two from an excess of heterozygotes (Table 2). Eleven of the 30 *F*_IS_ values at the six loci estimated for the five populations were significantly different from zero (*P* < 0.01). Significant deviations were not evenly distributed among samples or loci, nor were they associated with a particular locus or sample. DHWE was observed at one locus in WD, but at four loci in GJ.

In total, 233 different alleles were observed across all loci and all samples, ranging from 15 for the Hdh154 locus to 64 for the Hdh513 locus. No population had a diagnostic allele. In the five populations, 64.0 to 76.6% of the alleles were rare alleles with a frequency <5%. They were detected at most loci and were not associated with a particular locus in either population.

In testing the departure from mutation-drift equilibrium based on heterozygosity excess or deficiency for five populations, bottleneck analysis was conducted using Bottleneck software under the TPM of microsatellites. No population displayed significant heterozygosity excess (*P* > 0.05) through the sign test, standardized differences test and Wilcoxon sign rank test, suggesting that five hatchery populations have not experienced a recent bottleneck.

### 3.2. Genetic Differentiation among Populations

**Figure 2.**
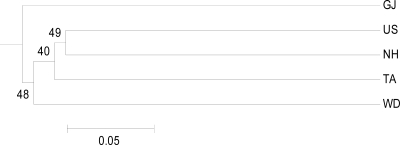
UPGMA dendrogram showing the phylogenic relationships among five cultured populations of the Pacific abalone. The abbreviations are as follow: US (Ulsan), GJ (Geoje), NH (Namhae), WD (Wando) and TA (Taean).

**Figure 3.**
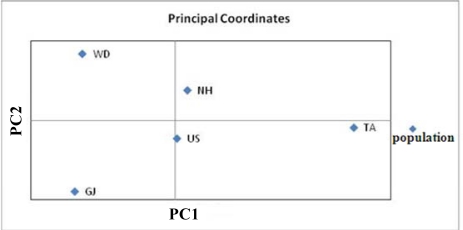
Principal components analysis, plotting the relationships of the studied five *H. discus hannai* cultured populations based on *D*_CE_ genetic distances [21]. The abbreviations are as follow: US (Ulsan), GJ (Geoje), NH (Namhae), WD (Wando) and TA (Taean).

Low but significant genetic differentiation (overall *F*_ST_ = 0.009, *P* < 0.01) was observed among populations, indicating that the level of genetic heterogeneity among them was low. Table 3 shows the pattern of genetic differentiation among populations observed by comparing *D*_CE_ and mean pairwise *F*_ST_ and *R*_ST_ values using composite allele frequency data. Values of pairwise *F*_ST_ among populations were significantly different from zero in all pairwise comparisons except one (all *P* < 0.01 after sequential Bonferroni correction, except for *P* = 0.086 between NH and US). On the other hand, the pairwise *R*_ST_ among populations was significantly different from zero in only five pairwise comparisons. Estimates of *F*_ST_ (range 0.0040 to 0.0176) were generally lower than *R*_ST_ values (range −0.0082 to 0.1016). The *D*_CE_ values were similar to the *F*_ST_ and *R*_ST_ values, in the range of 0.018 to 0.026. The AMOVA from the distance matrices for 233 individuals permitted partitioning of the overall variation. The amount of variation attributable to differences between individuals within populations and within-individual differences were large (12.09 and 87.06, respectively; *P* < 0.05), while only a low but significant proportion of variation (0.85%; *P* < 0.05) was detected among populations. The UPGMA tree and the PCA scatter plot constructed on the basis of the *D*_CE_ values showed that no population formed a separate cluster (Figures 2 and 3). The US population from the eastern coast and the NH population from the southern coast were the most closely related populations, while GJ from the southern coast was the most isolated from the others, the same groupings found with the PCA scatter plot analysis. Together, pairwise *F*_ST_ and *R*_ST_ tests, the UPGMA tree, and PCA on the five cultured populations of *H. discus hannai* did not show a consistent relationship between geographic and genetic distances.

## 4. Discussion

Although the two loci, Hdd114B and Hdd229, used in this study were originally described and designed for a congener (*H. discus discus*) [14], they all worked satisfactorily for *H. discus hannai*. This remarkable level of cross-species microsatellite conservation is a clear indication of the close molecular relatedness of the two species. Similar results were observed in the microsatellite analysis of *H. discus discus* [17,35].

The genetic variability of the six microsatellite loci proved to be extensive. Genetic variability in the five hatchery populations (average *N*_A_ = 23.47, average *H*_e_ = 0.904) was significantly higher than previously measurements from Japanese and Chinese hatcheries (average *N*_A_ = 5.43 to 8.7, average *H*_e_ = 0.575 to 0.774) [6,20]. However, Li *et al*. [6] reported that for the Japanese natural Pacific abalone populations, the average number of alleles per locus and heterozygosity was 22.4 and 0.793, respectively. Similar genetic variability were also detected for eight Korean natural Pacific abalone populations (average *N*_A_ = 28.13, average *H*e = 0.916; An *et al*., data not published). Most of microsatellite markers used in above studies were same; then, can be compared on an equal basis. These values are similar to our results, indicating that a high level of genetic variation has been preserved during the domestication of these five cultured Pacific abalone populations in Korea. The maintenance of genetic diversity in hatchery populations has been reported for other marine organisms. For example, no differences in genetic diversity could be detected between wild and hatchery populations of Pacific oysters in Australia and China [36,37]. The use of hundreds of parental males and females from different farms for spawning might contribute to the maintenance of genetic diversity in these animals.

Significant deviations from HWE were observed in 12 of the 30 population-locus cases. Ten cases had lower heterozygosity than predicted by HWE. Heterozygote deficiency has been reported for many other marine invertebrates [38,39]. However, in many cases, its cause remains unknown [40]. In the hatchery populations, heterozygote deficiency is commonly caused by a limited number of founders or founder effects. The observed *F*_IS_ in this study can attribute to the founder effect. The limited dispersal nature of abalones together with the sweepstake style of reproductive success may easily lead to such a non-random mating of population [35]. The heterozygote deficiency observed in hatchery populations is probably caused by unequal contributions of a limited number of pair mating to the next generation. Alternatively, the null alleles observed at the microsatellite loci present a likely explanation for the deviation [41]. In our study, null alleles were observed at five of the six loci, and no significant deviation from HWE was observed at the Hdd114B locus, which did not have any null alleles. In contrast, two of the 12 significant HWE deviations were caused by an excess of heterozygotes. Recent population bottlenecks, when a population of interest experiences a reduction in size, sometimes also reduce the number of homozygotes [42,43]. However, bottleneck analysis showed that five hatchery populations have not experienced a recent bottleneck; thus, the possibility of recent bottleneck seems unlikely. Alternatively, it is possible to ascribe the heterozygote excess in cultured stocks to an overdominance phenomenon, which reduces the survival of homozygotes [44]. Li *et al*. [6] and Hara and Sekino [19] observed, on the basis of microsatellite analysis, heterozygote excesses in 8 of 10 and 13 of 16 significant HWE deviations in Pacific abalone hatchery stocks, respectively.

Genetic differentiation detected by *F*_ST_ and *R*_ST_ values were low but significant beteen most pairs of hatchery populations. Although we cannot trace the origins of the significant differences between the hatchery populations with no records from the farms, this differentiation observed among cultured populations is likely be due to a low number of founding individuals in hatcheries, which increases the effect of genetic drift, as has previously been reported for other hatchery-reared species including abalone [6,19,45–47]. In addition, different selection procedures in hatchery practice may have also led to changes in the genetic composition of hatchery populations [20]. The AMOVA of all six microsatellites revealed that 0.85% (*F*_ST_ = 0.009, *P* = 0.040) of the genetic variance occurred among the hatchery samples, with the remainder of the variance occurring between individuals within populations and within individuals. To our knowledge, this is the lowest among-population variance component reported for this species. Previously reported *F*_ST_ values for hatchery Pacific abalone (*F*_ST_ = 0.243 to 0.427 at six polymorphic loci for three hatchery populations in Japan; *F*_ST_ = 0.056 to 0.259 at nine polymorphic loci for nine hatchery populations in Japan; *F*_ST_ = 0.0546 to 0.0976 at seven polymorphic loci for five hatchery populations in China) [6,19,20] all exceeded our estimated value. The UPGMA tree and the PCA scatter plot constructed using *D*_CE_ genetic distances did not indicate a regional structure, *i.e*., individuals from nearby regions were not grouped together (Figures 2 and 3). Although differences in the number of polymorphic loci and the number of populations examined in a study will influence *F*_ST_ values and some element of hatchery selection may have also led to changes in the genetic composition of the cultured populations, the maintenance of genetic diversity, the relatively low *F*_ST_ value and the lack of a relationship between the geographic and genetic distances suggest that some gene flow has occurred among populations during the extensive aquaculture of abalone, though the possibility of widespread exchanges of stocks and eggs between hatcheries by local farmers cannot be excluded.

## 5. Conclusions

In conclusion, an understanding of the genetic structure and diversity among marine resources, especially for species for which artificial stocks are produced by hatchery-produced seed for natural resource restoration, are critical for the establishment of suitable guidelines for resource management and selective breeding. No detailed information is available to date on the genetic diversity of hatchery populations of Pacific abalone in Korea. In this study, we reported that relatively high genetic variability and significant but minor genetic differentiation were detected among the hatchery populations of Pacific abalone in Korea based on analysis of six microsatellite loci. The lack of a relationship between the geographic and genetic distances implied extensive abalone aquaculture. The allelic composition and diversity of Pacific abalone should be carefully considered regarding the seedling and stocking practice of hatcheries in order to conserve the genetic diversity of the natural population. Such information will be of assistance in the genetic management of fisheries and the successful implementation of stock-enhancement programs.

## Figures and Tables

**Table 1.** Collection details for five hatchery-reared populations of *Haliotis discus hannai*.

**Population’s Name (Abbreviation)**	**Sample Locality**	**Sample Size**	**Collection Date**
Ulsan population (US)	Eastern Area (Ulsan); 35° 32′ N, 129° 25′ E	51	February. 2004
Geoje population (GJ)	Southern Area (Geoje); 34° 53′ N, 128° 41′ E	43	June. 2003
Namhae population (NH)	Southern Area (Namhae); 34° 42′ N, 128° 0′ E	49	June. 2003
Wando population (WD)	Southern Area (Wando); 34° 23′ N, 126° 51′ E	41	May. 2003
Taean population (TA)	Western Area (Taean); 36° 40′ N, 126° 16′ E	39	March. 2004

**Table 2.** Allelic variability observed at six microsatellite loci in five hatchery-reared populations of *Haliotis discus hannai*.

**Population (No.)**		**Microsatellite Loci**						
		**Hdh1321**	**Hdd114B**	**Hdd229**	**Hdh513**	**Hdh512**	**Hdh145**	**Mean**
**Ulsan (51)**	*N*_A_	29	26	22	39	31	10	26.17
	*A*_R_	27.59	24.07	20.40	34.45	28.70	9.23	24.07
	*S*	264–380	152–256	168–218	422.000	70–200	130–154	
	*U*	2	2	0	2	4	1	1.83
	*H*_e_	0.961	0.943	0.913	0.967	0.964	0.750	0.916
	*H*_o_	1.000	0.902	0.647	0.824	1.000	0.549	0.820
	*F_IS_*	−0.041	0.044	0.293*	0.149*	−0.037	0.270*	0.106
	*P*	0.934	0.727	0.000*	0.038	0.211	0.000*	

**Geoje (43)**	*N*_A_	29	23	21	31	23	10	22.83
	*A*_R_	28.02	22.05	20.42	29.83	22.52	9.71	22.09
	*S*	264–366	152–252	172–226	190–422	88–148	114–154	
	*U*	0	1	2	3	2	1	1.50
	*H*_e_	0.959	0.898	0.905	0.958	0.950	0.693	0.894
	*H*_o_	0.814	0.930	0.674	0.744	0.953	0.488	0.767
	*F_IS_*	0.153	−0.037	0.257*	0.225	−0.003	0.298*	0.143
	*P*	0.000*	0.940	0.000*	0.011	0.001*	0.000*	

**Namhae (49)**	*N*_A_	32	29	21	40	27	11	26.67
	*A*_R_	28.92	27.04	20.16	36.38	25.36	10.83	24.78
	*S*	288–398	154–258	164–218	174–422	74–142	106–152	
	*U*	6	1	1	8	1	3	3.33
	*H*_e_	0.925	0.951	0.945	0.968	0.950	0.777	0.919
	*H*_o_	0.571	0.918	0.857	0.735	0.878	0.551	0.752
	*F_IS_*	0.385*	0.034	0.094	0.243*	0.077	0.293*	0.184
	*P*	0.000*	0.485	0.128	0.001*	0.095	0.000*	

**Wando (41)**	*N*_A_	26	24	18	36	20	5	21.50
	*A*_R_	25.60	23.46	17.56	35.16	19.89	5.00	21.11
	*S*	288–378	152–270	168–224	182–422	74–132	130–140	
	*U*	2	4	1	2	0	0	1.50
	*H*_e_	0.945	0.930	0.879	0.969	0.934	0.651	0.885
	*H*_o_	0.951	0.927	0.683	0.976	0.951	0.537	0.838
	*F_IS_*	−0.007	0.004	0.225*	−0.007	−0.019	0.178	0.054
	*P*	0.157	0.764	0.000*	0.178	0.275	0.302	

**Taean (39)**	*N*_A_	28	19	18	31	19	6	20.17
	*A*_R_	28.00	19.00	18.00	31.00	19.00	6.00	20.17
	*S*	266–384	152–260	172–218	188–422	90–134	128–140	
	*U*	3	2	0	2	0	0	1.17
	*H*_e_	0.945	0.907	0.925	0.964	0.922	0.781	0.907
	*H*_o_	0.974	0.897	0.462	0.897	0.974	0.641	0.808
	*F_IS_*	−0.031	0.010	0.505*	0.070	−0.058*	0.181	0.111
	*P*	0.996	0.581	0.000*	0.170	0.000*	0.025	

***Mean all populations***								

	*N*_A_	28.80	24.20	20.00	35.40	24.00	8.40	23.47
	*A*_R_	27.63	23.12	19.31	33.36	23.10	8.15	22.44
	*U*	2.60	2.00	0.80	3.40	1.40	1.00	1.87
	*H*_e_	0.951	0.926	0.909	0.965	0.944	0.730	0.904
	*H*_o_	0.919	0.915	0.607	0.835	0.951	0.553	0.797

Number of samples (No.), number of alleles per locus (*N*_A_), allellic richness (*A*_R_), size in bp of alleles (*S*), number of unique alleles (*U*), expected heterozygosity (*H*e), observed heterozygosity (*H*o), inbreeding coefficient (*F*_IS_), and probability of significant deviation from Hardy-Weinberg equilibrium (*P*) are given for each population and locus. Wide significance levels were applied using the sequential Bonferroni technique (*k*= 6) [31].

* Significant at *P* < 0.01. Calculations assume that individuals with one microsatellite band are homozygous for the allele. Number in parentheses below *F*_IS_ indicates the probability of significant heterozygosity excess or deficit.

**Table 3.** *D*_CE_ distance (below the diagonal) and mean *F*_ST_ estimates and *R*_ST_ estimates (above the diagonal) between each pair of five hatchery-reared populations of *Haliotis discus hannai*.

**Population**	**Ulsan**	**Geoje**	**Namhae**	**Wando**	**Taean**
Ulsan	–	0.0068* (0.0744*)	0.0050* (0.0341^NS^)	0.0088* (0.0198^NS^)	0.0091* (0.0053^NS^)
Geoje	0.019	–	0.0115^NS^ (0.0289*)	0.0124* (0.0231^NS^)	0.0176* (0.1016*)
Namhae	0.018	0.024	–	0.0068* (−0.0082^NS^)	0.0098* (0.0524*)
Wando	0.019	0.021	0.020	–	0.0172* (0.0332*)
Taean	0.018	0.026	0.023	0.020	-

*D*_CE_ distance [32] and pairwise *F*_ST_ and *R*_ST_ [29,30] are measures of genetic distance and genetic differentiation between populations, respectively. Number in parenthesis indicates *R*_ST_. Wide significance levels were applied using the sequential Bonferroni technique (*k* = 10) [31].

* Significant at *P* < 0.01. NS is nonsignificant after sequential Bonferroni correction.
